# Intra-urban differentials in the exclusive use of hygienic methods during menstruation among young women in India

**DOI:** 10.1371/journal.pgph.0002047

**Published:** 2023-06-13

**Authors:** Aditya Singh, Mahashweta Chakrabarty, Rakesh Chandra, Sourav Chowdhury, Shivani Singh

**Affiliations:** 1 Banaras Hindu University, Varanasi, India; 2 Girl Innovation, Research, and Learning Centre, Population Council, New York, NY, United States of America; 3 Tata Institute of Social Sciences, Mumbai, India; 4 Raiganj University, Raiganj, India; 5 India Health Action Trust, Lucknow, India; University at Buffalo, UNITED STATES

## Abstract

Menstrual hygiene among women is a critical public health issue in urban India, but it remains understudied and under-researched. However, to our knowledge, no national level study in India has yet examined the differentials in the exclusive use of hygienic methods among young women (aged 15–24) in urban India. This study attempts to fill this gap by analysing biodemographic, socioeconomic, and geographic differentials in the exclusive use of hygienic methods among these women. We analysed data on 54561 urban women aged 15–24 from National Family Health Survey-5, 2019–21. We used binary logistic regression to examine differentials in the exclusive use of hygienic methods. To examine spatial variation, we mapped exclusive use of hygienic methods across Indian states and districts. The study found that two-thirds of young women in urban India reported exclusive use of hygienic methods. However, there was significant geographic heterogeneity observed at both state and district levels. In states such as Mizoram and Tamil Nadu, the use of hygienic methods was over 90%, while in Uttar Pradesh, Bihar, Chhattisgarh, and Manipur, it was less than 50%. The district-level variation in exclusive use of hygienic methods was even more striking. In many states, districts with extremely low exclusive use (less than 30%) were located in close proximity to districts with high exclusive use. Being poor, uneducated, Muslim, having no mass media exposure, living in the north and central regions, not having a mobile phone, getting married before 18, and having an early experience of menarche were associated with lower exclusive use of hygienic methods. In conclusion, substantial biodemographic, socioeconomic, and geographic differentials in the exclusive use of hygienic methods suggest the need for context-specific behavioural interventions. Mass media campaigns and targeted distribution of subsidized hygienic methods could help reduce the existing inequities in the exclusive use of hygienic methods.

## Introduction

Developing countries, including India, are witnessing rapid growth in their urban population. India’s urban population has increased from 17.0% in 1951 to 34.9% in 2020 (285 million), with an annual growth of 2.3% between 2015 and 2020 [1]. Although cities are engines of economic growth and bring significant improvements in urban residents’ health and living standards, they are also much more unequal than rural areas [2, 3]. Evidence suggests that an urban advantage in health and healthcare use exists when comparing averages of urban and rural areas as rural residents had lower health information access and use [4–7]. However, significant intra-urban heterogeneity is also revealed when examining these indicators across urban areas of a country [8, 9]. It has been evidenced that for some specific health outcomes and health services utilization indicators, certain urban population groups fare even more poorly than the rural population [10, 11]. However, government policies and programs often pay less attention to these intra-urban inequalities in health and healthcare use. Instead, their focus primarily remains on improving the health outcomes of rural communities. It might be owing to policymakers’ strong belief in the so-called ’urban advantage’ theory, which holds that urban areas have an advantage over rural areas in terms of health and wellbeing [7]. Thus, the issue of intra-urban differentials in health outcomes and healthcare use gets relegated to an issue of secondary concern.

Menstruation, a natural biological event signifying the start of a woman’s reproductive period, affects billions of girls and women worldwide [12, 13]. It is crucial to the dignity and wellbeing of women and girls to maintain adequate cleanliness during the period of menstruation in order to limit the risk of contracting reproductive tract infections [14, 15]. While some women and girls use tampons, sanitary pads, and menstrual cups, others use cloths, paper, ash, plant leaves, and many other materials to absorb menstrual blood and maintain personal cleanliness during menstruation [16, 17]. The first three methods of menstrual hygiene management (tampons, sanitary pads, menstrual cups) are commonly classified as ’hygienic’ in the previous studies and reports [18, 19]. Although the established classification scheme is suitable for analytical purposes, it has the potential limitation of not being able to capture the full range of women’s experiences and perspectives regarding menstrual hygiene. It must be emphasized here that the use of this classification in this paper is solely intended for analytical purposes and is not intended to stigmatize or marginalize women who use alternative menstrual hygiene products/methods. The authors are committed to using language that is precise, inclusive, and respectful of the diversity of women’s experiences with menstrual hygiene.

A growing body of evidence from low- and middle-income countries suggests that millions of women and girls resort to manage their periods with proper hygiene and dignity [20, 21]. This puts such women at a greater risk of reproductive tract infections, urinary tract infections, and other related problems [15, 22, 23]. Moreover, menstruation and menstrual hygiene-related insecurity contribute to school absenteeism and dropout risk for millions of girls and women [24–26]. The links between unmet menstrual health and hygiene requirements and RTIs and school absence are complex and mixed though [20, 27, 28]. Also, the shame and stigma attached to menstruation result in restrictions, prohibitions, and exclusion from public life, stopping women reach their full potential [29, 30]. It is therefore important that women are able to manage their menstruation with proper hygiene and dignity.

There is a significant body of literature documenting urban-rural dichotomy in the utilization of hygienic methods in developing countries, but little is known about the emerging heterogeneity in the use of hygienic methods within urban areas [15, 31–34]. In the Indian context, several small-scale studies based on individual cities have documented the utilization of hygienic methods during menstruation [35, 36]. A considerable amount of literature has been published to understand the knowledge, perceptions, and practices regarding menstrual hygiene, focusing on young women in India. However, they are mainly micro-level community-based studies limited to small geographical areas [31, 35–39]. Previous national-level studies in India have consistently shown that level of education, household wealth, mass media exposure, and place of residence are important determinants of the use of hygienic methods during menstruation [32, 33, 40, 41]. However, none has examined the factors affecting the exclusive use of hygienic methods among young urban women at the national level.

Several previous studies have reported that adolescent and young women in South Asian countries like India may find menstruation particularly challenging because of pervasive stigma [17, 42–46]. Most people consider menstruating women to be dirty or filthy [47, 48]. Having a menstrual period is stigmatising and restricts a woman’s ability to go about her daily life and keep herself clean [49–51]. Due to lack of autonomy, and disposable income menstruating adolescent girls and young women face evident risks to their health, safety, and quality of life when such limitations are imposed [20, 52]. The difficulties of period management in India can only be overcome if more is known about the menstrual hygiene practices of young women in the nation [44].

The urban population in Indian cities is socioeconomically and demographically quite diverse. Therefore, a crucial prerequisite for improving the utilization of safe and hygienic menstrual methods among young women in urban India is to ascertain the specific groups of women and girls who are at a disadvantage. Using the latest data available from the National Family Health Survey series, this study examines the biodemographic, socioeconomic and geographic differentials in the exclusive use of hygienic methods among women aged 15–24 years residing in urban India.

## Data and methods

**Fig 1:**
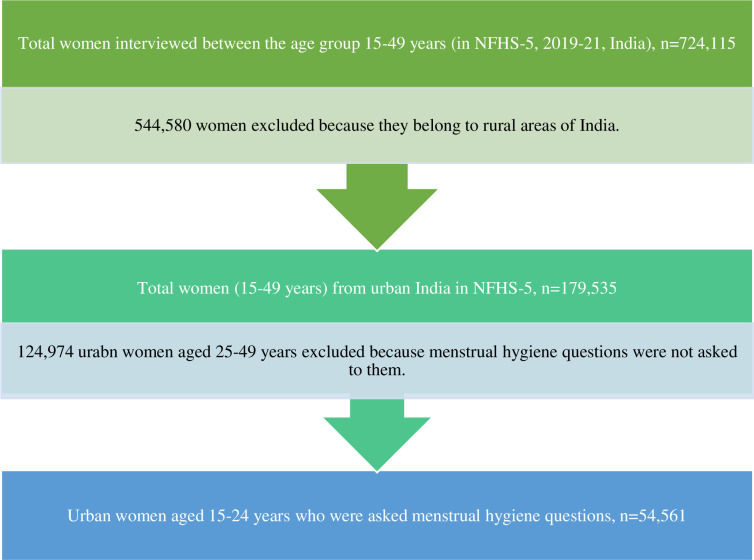
Flow chart showing the process of selection of urban women (15–24 years) sample for the current study.

This study used data from the National Family Health Survey (2019–2021). The NFHS is a nationally representative cross-sectional survey that collects data on various health-related issues, including maternal and child care, domestic violence, reproductive health and family planning [19]. The details of the sampling procedure and sample size are available in the national and state reports [19]. NFHS-5 interviewed 724,115 women of the reproductive age group (15–49 years) from 636,699 households in 28 states and 8 Union territories (UTs). In this paper, 54,561 urban women aged 15–24 from 28 states and 8 UTs were included in the analysis (see Fig 1 for the sample selection process).

### Conceptual framework

**Fig 2:**
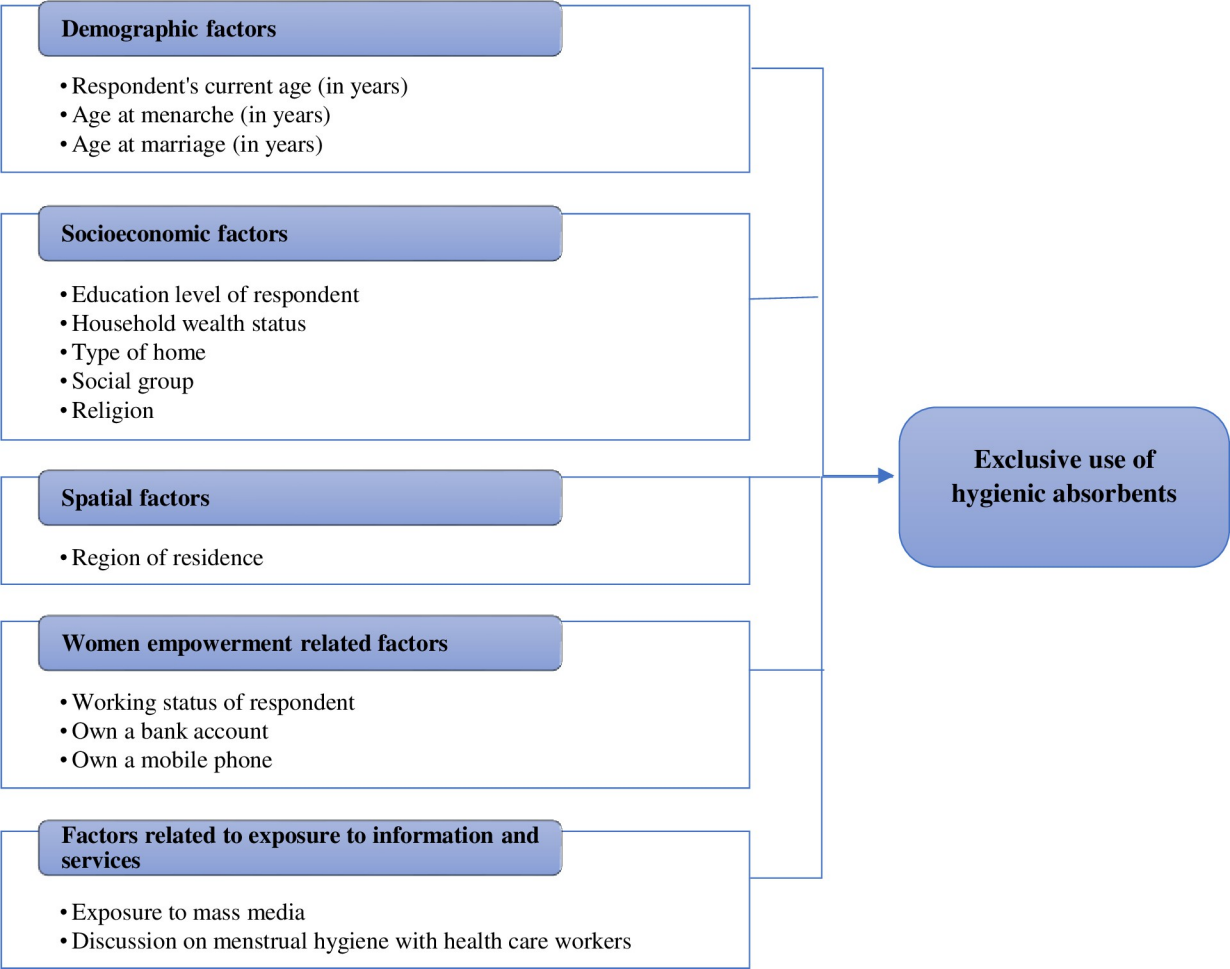
Conceptual framework: Factors affecting exclusive use of hygienic methods.

This study examines urban Indian women’s use of hygienic methods and influencing factors. A framework was developed using evidence from the existing literature on factors associated with using hygienic methods during menstruation [33, 41, 53–56] (see Fig 2). The framework showed pathways through which various factors might affect the exclusive use of hygienic methods among urban Indian women. It was hypothesized that the exclusive use of hygienic methods was affected by various factors: demographic factors, socioeconomic factors, geographic factors, and factors related to exposure to information and services. The list of variables considered for analysis is provided later in this section.

### Variables

#### Dependent variable

The NFHS-5 included a question with multiple responses to determine the methods of protection used by respondents to prevent bloodstains during their menstrual period. The question comprised seven response categories, including menstrual cups, locally made napkins, sanitary napkins, tampons, cloth, nothing, and others. For the purpose of analysis, these responses were grouped into two categories: a) menstrual cups, locally made napkins, sanitary napkins, and tampons, and b) cloth, nothing, and others. Based on these categories, a binary outcome variable was created with women who used materials included in category ‘a’ only considered as "exclusive users of hygienic methods" (coded as ‘1’). Women who used materials included in category ‘b’ or a combination of materials from both categories were grouped as "non-exclusive users of hygienic methods" (coded as ‘0’).

#### Independent variables

Several relevant socioeconomic and demographic predictors (including “respondent’s current age”, “age at menarche”, "age at marriage", "women’s education”, “respondents’ social group”, “religion”, “household wealth status”, “region of residence”, “types of home”, “exposure to mass media”, “discussion on menstrual hygiene with healthcare workers”, “respondent’s working status”, “ownership of a bank account”, and “mobile phone” were included in the analysis. The selection of variables was based on existing research on menstrual hygiene management and availability variables in the NFHS-5 dataset [11, 33, 32, 41, 53]. Table 1 describes the explanatory variables used in this study, their definitions and categories.

**Table 1:** Operational definition of variables used in the study.

Variables	Description
**Age at menarche (in years)**	Age at menarche is the age when a woman has her first menstrual period. For the purpose of this study, we divided it into four categories: "less than 12 years" (coded as 1), "13–15 years" (coded as 2), "more than 16 years" (coded as 3), and for those who could not remember their age at menarche, it was coded as "do not know" (coded as 0).
**Age at marriage (in years)**	Age at marriage was classified into three categories- ‘marriage before 18 years’ (coded as 0), ‘marriage after 18 years’ (coded as 1), and ‘not married’ (coded as 2)
**Education level of respondent**	The education level variable in this study represents the highest level of education attained by respondents, categorized into four groups: ’no education’ (coded as 0), ’primary’ (coded as 1), ’secondary’ (coded as 2), and ’higher’ (coded as 3).
**Household wealth status**	The wealth index measures the socioeconomic status of a household. It is a composite index of household amenities and assets. In NFHS-5, every household is given a score based on the number of consumer goods they own. A total of 33 assets and housing characteristics were taken into consideration to prepare a factor score using Principal Component Analysis. After that, this factor score is divided into five equal categories: ‘poorest’ (coded as 1), ‘poorer’ (coded as 2), ‘middle’ (coded as 3), ‘richer’ (coded as 4), and ‘richest’ (coded as 5), each with 20% of the population.
**Social groups**	The entire population of our study sample is divided into four social groups: ‘Scheduled Caste’ (coded as 1), ‘Scheduled Tribe’ (coded as 2), ‘Other Backward Classes’ (coded as 3), ‘Others’ (often referred to as General) (coded as 4).
**Religion**	For the purpose of the study, we have recoded religions into four categories–‘Hindu’ (coded as 1), ‘Muslim’ (coded as 2), ‘Christian’ (coded as 3), and ‘Others’ (coded as 4). Others include all religious groups other than Hindu, Muslim, and Christian.
**Region of residence**	To construct this variable, Indian states and UTs are grouped into six categories. ‘Northern’ (coded as 1) includes Jammu & Kashmir, Ladakh, Himachal Pradesh, Punjab, Rajasthan, Haryana, Uttarakhand, Chandigarh (Union Territory—UT) and Delhi; ‘central’ (coded as 2) includes the states of Uttar Pradesh, Madhya Pradesh and Chhattisgarh; ‘eastern’ (coded as 3) includes the states of Bihar, Jharkhand, West Bengal and Odisha; ‘western’ (coded as 4) includes the states of Gujarat, Maharashtra, Goa and UTs of Dadra & Nagar Haveli and Daman & Diu; ‘southern’ (coded as 5) includes the states of Kerala, Karnataka, Andhra Pradesh, Tamil Nadu and the UTs of Andaman & Nicobar Islands, Pondicherry and Lakshadweep); ‘northeastern’ (coded as 6) includes the states of Sikkim, Assam, Meghalaya, Manipur, Mizoram, Nagaland, Tripura, and Arunachal Pradesh. This classification has been used by many previous studies [19]
**Type of home**	Based on the woman’s relationship with the household head, the type of home in which she resided was categorized as a ‘marital home’ (coded as 1) (wife, daughter-in-law, or sister-in-law of the household head), ‘natal home’ (coded as 2) (daughter, granddaughter, or niece of the household head), or ‘other’s home’ (coded as 3) (e.g., non-relatives such as domestic servants working in the household, orphans, deserted young women).
**Working status of the respondent**	Working status indicates the employment condition of the respondent. A dichotomous variable is formed: ‘not working’ (coded as 1) and ‘working’ (coded as 2)
**Exposure to mass media**	Three questions were asked to women in the NFHS-5 survey. They are i) how often they read newspapers/magazines, ii) how often they watch television, and iii) how often they listen to the radio. The responses are ‘almost every day’, ‘at least once a week’, ‘less than once a week’, and ‘not at all’. Based on these responses, a composite index is computed, and divided into four categories: ‘no exposure’ (coded as 0) if the respondent is not exposed to any mass media; ‘low exposure’ (coded as 1) if a respondent is exposed to any one type of mass media; ‘medium exposure’ (coded as 2) if the respondent is exposed to any two types of mass media; ‘high exposure’ (coded as 3) if the respondent is exposed to all three types of mass media.
**Discussed menstrual hygiene with healthcare workers (in last three months)**	Questions were asked to the respondents in NFHS-5 are- i) in the last three months, if the respondent has met with any health worker- including an auxiliary nurse midwife (ANM), accredited social health activist (ASHA), *Anganwadi* worker (AWW), also known as Integrated Child Development Services worker, multipurpose worker (MPW), or any other community health worker; and ii) if they have discussed menstrual hygiene during the meeting. If the respondent did not discuss menstrual hygiene with healthcare workers, they are coded as 0; if discussed, 1.
**Own a bank account**	Whether a respondent owns a bank/savings account by herself–‘yes’ (coded as 1); ‘no’ (coded as 0)
**Own a mobile phone**	Whether a respondent owns a mobile phone by herself–‘yes’ (coded as 1); ‘no’ (coded as 0)

### Statistical analysis

The analysis starts with examining background characteristics of young women (aged 15–24 years) in urban India sampled in the NFHS-5. Subsequently, bivariate analysis was performed to scrutinize the disparity in the dependent variable across the respondents’ demographic attributes and geographical factors. Thereafter, the multivariable binary logistic regression model was utilized to identify the correlates of the exclusive utilization of hygienic methods. This particular model was deemed appropriate for analysis since the response variable was dichotomous in nature [57]. Prior to conducting the multivariable binary logistic regression analysis, we first performed a Chi-squared test to evaluate the association between the outcome and each predictor variable. This initial step allowed us to eliminate any independent variables that did not demonstrate a statistically significant bivariate relationship with the outcome. All predictors that yielded a statistically significant result (p<0.05) in the Chi-squared testing were kept in the multivariable binary logistic regression. Given that our study involved several explanatory variables that could potentially exhibit correlation with one another, we also conducted an assessment of multicollinearity using variance inflation factors (VIF). The results of the logistic regression analysis were presented as odds ratios, accompanied by their corresponding p-values and 95% confidence intervals. All statistical analyses and modelling procedures were performed using Stata16 software [58]. In addition, ArcMap 10.5 software was used to create maps depicting the geographical distribution of the outcome variable at the state and district levels [59].

## Results

### Respondent characteristics

This study sample includes 54,561 urban 15-24-year-old women. Two-thirds of the sampled women were unmarried (see Table 2). Most women experienced menarche between 13–15. Seventy percent of females identified as Hindu. Females from the Other Backward Classes made up almost half of the sampled population (OBCs). Around 8% of women had no exposure to mass media. About 45% of the respondents lived in India’s north and central regions.

**Table 2:** Percentage distribution of urban women aged 15–24 years by background characteristics, NFHS-5 (2019–21), India.

Background characteristics	N (54,561)	%
**Respondent’s current age (in years)**		
15–19	26,929	49.36
20–24	27,632	50.64
**Age at menarche (in years)**		
Do not remember	369	0.68
≤12	10,871	19.92
13–15	41,200	75.51
≥16	2,121	3.89
**Age at marriage (in years)**		
Not married	40,359	73.97
<18 years	3807	6.98
≥18 years	10379	19.02
Do not know	16	0.03
**Respondent’s highest level of education**		
No education	1,826	3.35
Primary	2,206	4.04
Secondary	35,437	64.95
Higher	15,092	27.66
**Religion**		
Hindu	38,368	70.32
Muslim	10,564	19.36
Christian	3,573	6.55
Others	2,056	3.77
**Social Group**		
Do not know	3,176	5.82
Scheduled Caste	10,492	19.23
Scheduled Tribe	5,794	10.62
Other Backward Classes	22,270	40.82
Others	12,829	23.51
**Household wealth status**		
Poorest	12,857	23.56
Poorer	12,277	22.5
Middle	10,985	20.13
Richer	10,006	18.34
Richest	8,436	15.46
**Region of residence**		
North	12,893	23.63
Central	11,548	21.17
East	6,612	12.12
West	6,990	12.81
Southern	10,724	19.66
North-east	5,794	10.62
**Type of home**		
Marital	12,040	22.07
Natal	41,870	76.74
Other	240	0.44
Head of the household	411	0.75
**Exposure to mass media**		
No exposure	4,499	8.25
Low exposure	23,852	43.72
Medium exposure	22,224	40.73
High exposure	3,986	7.31
**Discussed menstrual hygiene with health worker**		
No	53,922	98.83
Yes	639	1.17
**Working status of respondent**		
Question not asked	46,390	85.02
Not working	7,148	13.1
Working	1,023	1.87
**Owns a bank account**		
Question not asked	46,390	85.02
No	2,107	3.86
Yes	6,064	11.11
**Owns a mobile phone**		
Question not asked	46,390	85.02
No	3,272	6
Yes	4,899	8.98

Note: N = No. of sample size.

### Prevalence of hygienic methods use by background characteristics

Table 3 presents the proportion of urban women exclusively using hygienic methods by their background characteristics. The exclusive use of hygienic methods is comparatively higher among urban women who experienced menarche after the age of 16, than among those who experienced menarche earlier. The use of exclusive hygienic methods was relatively higher among those who were either unmarried (71.0%) or married after the legal age of 18 years (63.9%) than those who were married before the legal age (52.3%).

**Table 3:** Percentage of urban women aged 15–24 who reported exclusive use of hygienic methods for menstrual bloodstain prevention, by selected background characteristics, NFHS-5 (2019–21), India.

Background characteristics	Percent of Women Using Hygienic methods (Weighted Percentage) N = 54,561	P value	95% CI	
			Lower	Upper
**Respondent’s current age (in years)**	(16.70)	0.004		
15–19	68.93		67.75	70.09
20–24	67.30		66.21	68.37
**Age at menarche (in years)**	(174.18)	<0.001		
≤12	69.17		67.57	70.72
13–15	67.95		66.86	69.03
≥16	70.39		67.26	73.34
**Age at marriage (in years)**	(731.06)	<0.001		
Not marriage	70.98		69.94	72.00
<18 year	52.36		49.80	54.90
≥18 year	63.93		62.41	65.43
**Respondent’s highest level of education**	(2753.57)	<0.001		
No education	33.42		29.78	37.27
Primary	39.5		36.21	42.88
Secondary	66.82		65.71	67.91
Higher	78.98		77.87	80.04
**Religion**	(1046.95)	<0.001		
Hindu	70.32		69.29	71.32
Muslim	56.31		54.05	58.54
Christian	80.37		77.08	83.29
Others	83.79		80.86	86.34
**Social groups**	(388.24)	<0.001		
Scheduled Caste	65.64		63.86	67.37
Scheduled Tribe	62.79		59.44	66.02
Other Backward Classes	65.70		64.35	67.02
Others	74.33		72.60	75.99
**Household wealth status**	(4080.84)	<0.001		
Poorest	48.04		46.11	49.98
Poorer	64.21		62.58	65.80
Middle	70.84		69.32	72.32
Richer	77.44		76.00	78.82
Richest	86.02		84.76	87.18
**Region of residence**	(2976.82)	<0.001		
North	77.97		76.67	79.23
Central	50.9		49.00	52.80
East	62.71		59.93	65.42
West	74.06		71.63	76.36
South	77.90		76.28	79.44
North-east	56.79		53.17	60.34
**Type of home**	(389.31)	<0.001		
Marital	60.98		59.45	62.49
Natal	70.24		69.19	71.27
Other	77.89		68.29	85.21
Head of the household	68.46		60.6	75.40
**Exposure to mass media**	(1820.67)	<0.001		
No exposure	44.79		42.06	47.55
Low exposure	64.35		63.04	65.63
Medium exposure	75.15		74.07	76.21
High exposure	74.82		72.32	77.17
**Discussed menstrual hygiene with health worker**	(4.55)	0.182		
No	68.05		67.05	69.02
Yes	71.85		66.19	76.89
**Working status of the respondent**	(2.85)	0.620		
Not working	67.43		65.31	69.48
Working	66.62		61.62	71.27
**Owns a bank account**	(72.4)2	<0.001		
No	60.06		56.60	63.43
Yes	69.92		67.73	72.01
**Owns a mobile phone**	(179.59)	<0.001		
No	59.03		56.04	61.96
Yes	73.08		70.92	75.12

Note: The values in parenthesis are Chi-squared statistics. CI = confidence interval, N = sample size

The exclusive use of hygienic methods among those having higher education was several times higher (about 79.0%) than those who never went to school (33.4%). It is observed that the exclusive use of hygienic methods was considerably higher among Christians (80.4%) than Hindus (70.3%) and Muslims (56.3%). The exclusive use of hygienic methods was lowest among the ST women (62.9%) and highest among the general (Others) category women (74.3%). As wealth status increased, there was a corresponding increase in the exclusive use of hygienic methods. While the proportion of women using hygienic methods was 86.0% in the wealthiest household, the proportion of such women in the poorest household was merely 48.0%.

Exclusive use of hygienic methods was most prevalent among women who reported medium exposure to mass media (75.2%). It was the lowest among those who reported no exposure to mass media (44.8%). The use of exclusive hygiene methods was slightly higher among those women who met health workers and discussed menstrual hygiene with them in the three months preceding the survey than those who did not meet any healthcare worker (71.9% vs 68.1%.). Women who owned bank accounts and mobile phones showed higher use of hygienic methods (70.0% and 73.1%, respectively) than those who did not. The prevalence was higher in the southern (78.0%) and northern regions (78.0%) of India as compared to the central and north-eastern regions (50.9% and 56.8%, respectively). The results also show significant diversity in the exclusive use of hygienic methods at the regional, state, and district levels (described in the next section).

### Spatial pattern of exclusive use of hygienic methods

**Fig 3:**
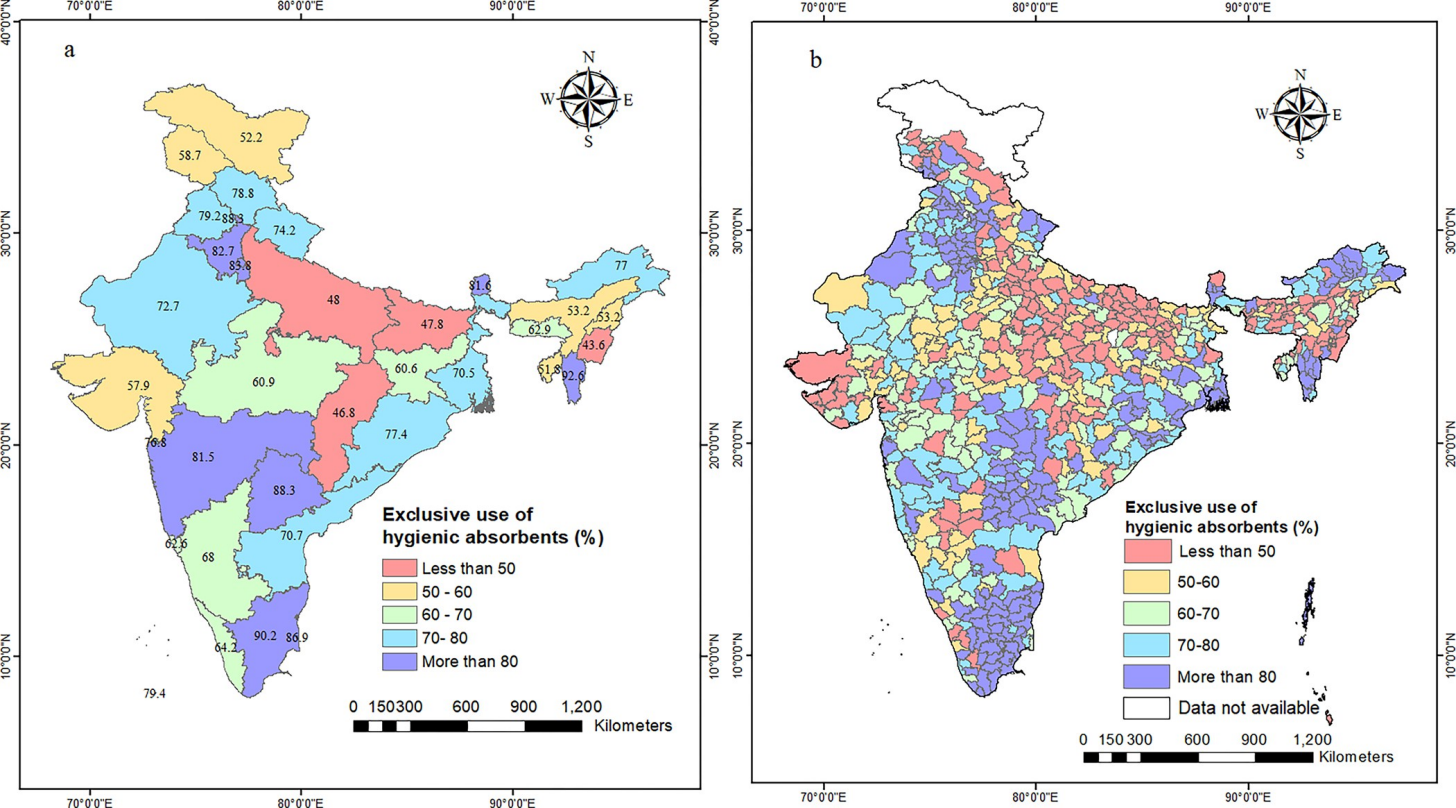
Distribution of exclusive use of hygienic methods during menstruation among urban women aged 15–24 years in India, NFHS-5, 2019–21. (a) State-wise distribution of exclusive use of hygienic methods during menstruation among urban women aged 15–24 years in India. (b) District-wise distribution of exclusive use of hygienic methods during menstruation among urban women aged 15–24 years in India. Source: authors’ own creation.

Analysis at the regional level provides only a broad idea regarding spatial variation in the exclusive use of hygienic methods and masks spatial heterogeneity at micro levels. Therefore, we mapped the exclusive use of hygienic methods at the state and district levels. Fig 3A presents the state-wise exclusive use of hygienic methods among young women in urban India. It is observed that the exclusive use was highest in Mizoram (92.6%), followed by Tamil Nadu (90.2%). On the other hand, it was lowest in Manipur (43.6%), Chhattisgarh (46.8%), Bihar (47.8%), and Uttar Pradesh (48.0%).

The state average obscures variation between individual districts within a state. Therefore, we analysed the patterns at the district level. Fig 3B depicts spatial patterns of exclusive use of hygienic methods among young urban women across all 707 districts of India. The spatial pattern of exclusive use of hygienic methods is considerably more varied than the state-level geographical pattern. The exclusive use of hygienic methods during menstruation varied among young urban women in different districts of India, with lower use reported in Kaimur district of Bihar (less than 15%) and higher use reported in Siddipet district of Telangana (more than 90%). The examination of within-state district-level patterns has revealed considerable between-district variation in the exclusive employment of hygienic methods across numerous states. For instance, in Chhattisgarh, the proportion of women reporting exclusive use of hygienic methods ranged from 26.0% in Mahasamund to 76.6% in Narayanpur. In Bihar, it varied from 11% in Kaimur to 78.8% in Madhepura, with less than 30% of exclusive use in five out of 38 districts in Bihar. Even in states with high exclusive use, such as Tamil Nadu, there were multiple districts where the prevalence was below 70%. In Karnataka, the exclusive usage ranged from 27.3% in Gadag and 33.3% in Bagalkot to 93.7% in Dakshina Kannada. In a similar vein, in Kerala, where the state average was 64.2%, the exclusive usage ranged from 35.9% in Idukki to 95.3% in Pathanamthitta.

In almost a quarter of all districts (175 out of 707), the exclusive use of hygienic methods was less than 50%. Several pockets of low exclusive use of hygienic methods were identified. The first pocket was spread over large swathes of central Indian states of Uttar Pradesh, Madhya Pradesh, Bihar, and Chhattisgarh. The second pocket was located in northeast India, comprising the districts of Assam, Manipur, Nagaland, and Meghalaya. Other pockets of low exclusive use were in western Gujarat, northern Kerala, northern Karnataka, and south-eastern Rajasthan. Within these pockets of low exclusive use, there were several districts with ultra-low exclusive use (less than 30%)—for example, Hardoi (18%), Ballia (21.3%), Lakhimpur (23.5%) districts of Uttar Pradesh and Kaimur (11%) and Gopalganj (23.8%) in Bihar, and Khandwa (17%) in Madhya Pradesh.

A little over one-fourth of all districts in the country (187 out of 707) had a prevalence of over 80%. Of these, about 10% (70 districts) had a prevalence of over 90%. There were three main pockets of high exclusive use of hygienic methods in the country, two in south India and one in northwest India. The southern pockets were spread over the district of Tamil Nadu and Telangana. The north-western pocket included almost all the districts of Delhi, Haryana and Punjab, and some southern Himachal Pradesh and northern Rajasthan districts. A small pocket of high exclusive use was also located in northern Odisha.

### Results of multivariable logistic regression

Table 3 presents the results of a Chi-squared test that investigated the relationship between exclusive use of hygienic methods and various independent variables. The findings indicated that variables such as the "working status of the respondents" and "discussion on menstrual hygiene with healthcare workers" did not demonstrate statistical significance and were, therefore, excluded from the multivariable binary logistic regression model. All other variables that were statistically significant (p ≤ 0.05) in the Chi-squared test were included in the model. Additionally, we calculated the Variance Inflation Factors (VIFs) to detect multicollinearity among the independent variables incorporated in the regression model. The mean VIF in our study was 2.8, which falls within the acceptable range of less than 10; therefore, all variables were retained in the final model [60] (The VIF values are available on request).

Table 4 presents adjusted odds ratios obtained from the multivariable logistic regression model. The results revealed that the odds of exclusive use of hygienic methods among those women who attained menarche at 16 or later were 22% higher (AOR: 1.22, 95% CI: 1.10–1.37) than those who attained it at 12 or earlier. Further, the results show that the odds of using hygienic methods among those who women who were married off before the legal age of 18 years were about 30% lower (AOR: 0.70, 95% CI: 0.63–0.78) than unmarried women.

**Table 4:** Logistic regression analysis showing the factors associated with the exclusive use of hygienic methods among urban women aged 15–24 years in India NFHS-5 (2019–21).

Background characteristics	Adjusted odds ratio	P value	95% CI	
			Lower	Upper
**Respondent’s current age (in years)**				
15–19 (Reference)				
20–24	0.82	<0.001	0.78	0.86
**Age at menarche (in years)**				
≤12 (Reference)				
13–15	1.07	0.012	1.01	1.12
≥16	1.22	<0.001	1.10	1.37
**Age at marriage (in years)**				
Not marriage (Reference)				
<18 year	0.70	<0.001	0.63	0.78
≥18 year	0.85	<0.001	0.77	0.93
**Respondent’s highest level of education**				
No education (Reference)				
Primary	1.28	<0.001	1.12	1.47
Secondary	2.18	<0.001	1.96	2.43
Higher	3.02	<0.001	2.68	3.40
**Religion**				
Hindu (Reference)				
Muslim	0.61	<0.001	0.58	0.64
Christian	1.34	<0.001	1.20	1.49
Others	1.22	0.001	1.09	1.37
**Social groups**				
Scheduled Caste (Reference)				
Scheduled Tribe	1.04	0.412	0.95	1.13
Other Backward Classes	0.91	0.001	0.86	0.96
Others	1.20	<0.001	1.12	1.27
**Household wealth status**				
Poorest (Reference)				
Poorer	1.65	<0.001	1.57	1.75
Middle	2.14	<0.001	2.02	2.27
Richer	2.83	<0.001	2.65	3.03
Richest	4.38	<0.001	4.05	4.74
**Region of residence**				
Central (Reference)				
North	2.41	<0.001	2.27	2.56
East	2.09	<0.001	1.95	2.24
West	1.68	<0.001	1.57	1.80
South	3.15	<0.001	2.96	3.35
North-east	1.84	<0.001	1.69	2.01
**Type of home**		<0.001		
Marital (Reference)				
Natal	0.93	0.103	0.85	1.02
Other	0.92	0.585	0.68	1.25
Head of the household	1.35	0.010	1.07	1.69
**Exposure to mass media**				
No exposure (Reference)				
Low exposure	1.29	<0.001	1.20	1.38
Medium exposure	1.34	<0.001	1.24	1.44
High exposure	1.24	<0.001	1.12	1.37
**Owns a bank account**		<0.001		
No (Reference)				
Yes	1.05	0.368	0.94	1.18
**Owns a mobile phone**				
No (Reference)				
Yes	1.25	<0.001	1.12	1.39

Note: CI = Confidence interval

The odds of exclusive use of hygienic methods varied considerably by the level of respondents’ education. The odds of exclusive use among women with secondary education and higher education were two (AOR: 2.18, 95% CI: 1.96–2.43) and three times (AOR: 3.02, 95% CI: 2.68–3.40) higher than those with no education. The odds among Muslim women were 40% lower (AOR: 0.61, 95% CI: 0.58–0.64 than their Hindu counterparts. The odds of using hygienic methods were 20% higher among others (AOR: 1.20, 95% CI: 1.12–1.27) than they are among SCs.

The wealth status exhibits a positive effect on the exclusive use of hygienic methods among urban women. The odds of exclusive use among women from the richest wealth quintile were nearly four times (AOR: 4.38, 95% CI: 4.05–4.74) higher than those from the poorest wealth quintile. The odds also varied significantly across the regions of India. For example, women from the south (AOR: 3.15, 95% CI: 2.96–3.35) and the north regions (AOR:2.41, 95% CI: 2.27–2.56) had higher odds of exclusive use of hygienic methods than women from the central region.

The odds of exclusive use among women who were the head of the households were 35% higher than those who resided in their marital homes (AOR: 1.35, 95% CI: 2.27–2.56). The odds of using hygienic methods among urban women living in their marital and natal homes were not significantly different. However, the odds for women with low, medium, and high mass media exposure were around 1.3 times higher than those without mass media exposure.

## Discussion

This paper aimed to identify the differentials and correlates of the exclusive use of hygienic methods during menstruation among young women in urban India. Two out of every three young women in urban India reported exclusive use of hygienic methods during menstruation. However, there was significant geographic heterogeneity in the exclusive use of hygienic methods at both state and district levels. The findings of this study revealed the existence of disadvantaged subgroups among urban women who require targeted interventions from policymakers through the implementation of context-specific programs and policies. The results highlighted that women’s education, mass media exposure, household wealth, and religion were positively linked to the exclusive use of hygienic methods during menstruation among urban women. Additionally, the analysis uncovered statistically significant associations between the exclusive use of hygienic methods and biodemographic factors such as age, age at menarche, and age at marriage.

In the current study, the exclusive use hygienic methods during menstruation were higher among younger women (aged 15–19) than their older counterparts (aged 20–24). One possible explanation is the proliferation of programs that offer teenage girls subsidized or free access to hygienic methods while they are still in school [61–63]. The prevalence of using hygienic methods increases with the rising age of menarche and marriage because women become increasingly aware of the various hygienic methods available to them as they age. A previous study reported that women having menarche at a younger age had insufficient knowledge of menstruation and the importance of using hygienic methods [64].

The findings of the current study suggest that urban women with higher education had a higher propensity to use hygienic methods exclusively than illiterate women. This finding is in line with existing evidence on the subject [18, 32, 41]. Women with a higher level of education are more cautious of the health risks of unhygienic menstrual practices, have more decision-making autonomy, and are often financially more independent [32, 53]. The education also provides opportunities to learn about reproductive health through exposure to mass media [11]. Consistent with prior research, Muslim women demonstrated a lower propensity to use hygienic methods than other religions, possibly due to a lack of mobility rights, limited information and awareness, and religious taboos on the disposal and storage of hygienic methods in the Muslim population [65].

The disparity in the exclusive use of hygienic methods across different economic/wealth groups has been a concern of many researchers. Several studies have documented the fact that household wealth has a positive effect on the exclusive use of hygienic methods [32, 33, 65]. The study affirms that young women in urban India exhibit similar trends, with those from the wealthiest households having four times greater odds of using hygienic methods compared to those from the poorest households. It has been evidenced that wealthier urban women have enough resources to buy hygienic methods. In contrast, poor women are generally less educated, often unemployed or stay-at-home mothers, and may struggle to afford the more expensive hygienic methods [33].

It must be noted here that one-third of the poor urban population in India resides in slums [66]. Lack of adequate housing and essential public amenities are the issues that have disproportionately affected the poor slum-dwellers [67, 68]. The inability to properly manage menstruation is a challenge for women and girls living in urban slums due to the factors mentioned above, as well as the lack of privacy, unsafe environments, vulnerability, inadequate infrastructure and services, poor water, sanitation and hygiene (WASH) facilities, and inadequate knowledge and practice of menstrual hygiene [69, 70]. Understanding the challenges women and girls face and enabling gender-sensitive policy and programme interventions that address their sanitation and hygiene needs is particularly timely in light of the poor living and sanitation conditions in slum areas and the current policy focus on WASH [70].

The present study revealed that increased mass media exposure increased the exclusive use of hygienic methods. This finding is consistent with several prior Indian and international investigations [32, 39]. The mass media could serve as reliable sources of information, enhancing women’s understanding of the health benefits of using hygienic methods and raising awareness of those available at subsidized prices [33, 53].

In this study, the ownership of mobile phones was positively associated with the exclusive use of hygienic methods during menstruation. The greater availability of mobile phones has brought about several advantages and opportunities for young women today. These mobile phones can potentially improve access to healthcare and understanding of the importance of menstrual hygiene and general wellbeing. Previous studies have also linked mobile phone ownership with digital awareness and increased use of exclusive hygienic methods [41].

State and district-level variations in the exclusive use of hygienic methods during menstruation among young urban women must be considered during policymaking and programme implementation. A possible explanation for this district-level variation could be the implementation of various subsidized and free sanitary napkin distribution initiatives. For example, the state of Tamil Nadu, where exclusive use of hygienic methods is over 80%, has provided 20 sanitary napkins free of cost to peri-urban women since 2011 under a scheme called *Pudhu Yugam* (New Era) [71]. The *’Swechha’* (Freedom) scheme of the Government of Andhra Pradesh provides free and subsidized sanitary napkins to girls in classes seven through ten. *Ruthu Prema* (Safe Periods) is a new program in Telangana that encourages using menstruation cups and provides free sanitary pads to all women via Anganwadi workers and municipal officers. Other states with high exclusive use of hygienic methods, such as Maharashtra (*Asmita*) and Punjab (*Udaan*), have also instituted free or subsidized sanitary napkin distribution programs. Moreover, in some areas of these states, schools have placed sanitary napkin vending machines in conjunction with local NGOs, dispensing locally produced napkins at a discounted rate [72].

Kerala (She Pad), Karnataka *(Suchi)*, Gujarat (*Taruni Suvidha)*, Odisha *(Khushi)*, and Uttarakhand (*Sparsh*) have also launched subsidized sanitary napkin schemes to promote the use of hygienic methods during menstruation among young girls and women [73–75]. However, it is unclear why some districts’ exclusive use of hygienic methods is substantially lower than other districts in these states. Further research is needed to unearth the reasons behind such within-state spatial disparities in the exclusive use of hygienic methods during menstruation among young urban women in these states.

Rajasthan (*Udaan*), Bihar (*Kanya Utthan Yojana*), Uttar Pradesh (*Kishori Suraksha Yojana*), Madhya Pradesh (*Udita*), Chhattisgarh (*Suchita*) Assam, Tripura (*Kishori Suchita Abhiyan*) and Manipur (*My Pad My Right*), also have implemented schemes to promote sanitary napkin use during menstruation [76–79]. In spite of this, a small percentage of young urban women in these states exclusively use hygienic methods during menstruation. It is most likely because these initiatives and programs run into several challenges, including supply and procurement problems, low-quality hygienic methods, a lack of knowledge and comprehension of the programs, financial irregularities, greater costs, and widespread corruption [80–82]. These challenges, however, are mainly organizational and can be dealt with by some structural changes.

The Government of India has also made several efforts to promote menstrual hygiene management among urban women via National Health Mission sponsored programmes. For example, the Central Government has set up a network of over 8500 subsidized pharmacies known as *Jan Aushadhi Kendras* (JAK) in all 640 districts [83]. However, this is insufficient for a population of 1.4 billion. In 2020, the government launched a brand of sanitary napkins known as *Suvidha* (Convenience), composed entirely of biodegradable materials and sold at a discounted price at these pharmacies [13, 84]. These programmes, however, have been hampered by procurement and supply issues, high costs, and a lack of an effective distribution mechanism [85]. These factors could be some of the reasons why the use of sanitary methods among young urban women in the vast majority of the country’s districts continues to remain lower than expected [86].

This study sheds light on the exclusive use of hygienic methods during menstruation among young urban women in India, but there are several limitations that need to be considered. Firstly, as this is a cross-sectional study, the causal relationship between predictor and outcome variables cannot be established. Moreover, the analysis solely focuses on demand-side factors, neglecting the potential impact of supply-side variables on the use of menstrual products. The absence of supply-side variables in the NFHS dataset means that factors such as the availability and pricing of hygienic methods at urban pharmacies and provision stores, as well as the provision of sanitary napkins in schools, could not be incorporated into the analysis, potentially influencing the study’s results. Additionally, the NFHS does not provide any information on the unmet need of menstrual materials or access to menstrual products in India. Further investigation is necessary to uncover the unmet needs of menstrual materials. Also, the survey does not provide any information on the materials used in locally prepared napkins. Furthermore, the NFHS data does not provide any information on whether women are properly sanitizing or washing their reusable menstrual hygiene materials, making it difficult to determine whether they are using such materials in a hygienic manner. The survey also does not cover critical factors such as social taboos, cultural norms and traditions, and disposability, which could potentially impact the use of hygienic methods [87]. Therefore, further research is needed to examine these factors and establish how they affect the use of hygienic methods. Furthermore, due to data constraints, this study does not include slum and non-slum variable in the analysis to determine intra-urban differences. Finally, the reasons behind the low exclusive use of hygienic methods in certain districts of some states with high averages remain unclear, and more investigation is needed to uncover these reasons. Overall, while this study provides valuable insights, these limitations highlight the need for further research to fully understand the factors influencing the use of menstrual hygiene materials among women in India.

## Conclusion

This study highlights the existence of significant socioeconomic, biodemographic, and geographic disparities in the use of hygienic menstrual methods among young women in urban India. To address these disparities, there is a need for advocacy campaigns, mass media exposure, educational outreach, and subsidized or free sanitary napkins for urban women, particularly those from underserved castes, tribes, and religions. Although there have been recent initiatives by central and state governments to improve access to hygienic menstrual products, many of these programs remain in the pilot phase or are limited to certain areas. Therefore, it is crucial to expand these initiatives to reach as many underserved individuals as possible. Additionally, governments must acknowledge the micro-level (district) disparities in the use of hygienic menstrual methods among urban women and focus on specific geographies highlighted in this study.

## References

[1] United Nations, Department of Economic and Social Affairs, Population Division (UN-DESA). World Urbanization Prospects. Demogr Res. 2018. Available: https://www.un.org/development/desa/publications/2018-revision-of-world-urbanization-prospects.html

[2] United Nations. Urbanization: expanding opportunities, but deeper divides. Department of Economic and Social Affairs News. 2020. Available: https://www.un.org/development/desa/en/news/social/urbanization-expanding-opportunities-but-deeper-divides.html

[3] McMichael AJ. The urban environment and health in a world of increasing globalization: issues for developing countries. Bull World Health Organ. 2000;78: 1117–1126. Available: https://www.ncbi.nlm.nih.gov/pmc/articles/PMC2560839/ 11019460 PMC2560839

[4] Chen X, Orom H, Hay LJ, Erika AW, Schofield E. Differences in Rural and Urban Health Information Access and Use. J Rural Heal. 2019;35: 405–417. doi: 10.1111/jrh.12335 30444935 PMC6522336

[5] Naik A. Urban-rural divide in healthcare infrastructure and planning for adaptability. Governance Now. 2021. Available: https://www.governancenow.com/views/columns/urbanrural-divide-in-healthcare-infrastructure-and-planning-for-adaptability

[6] Barik D, Thorat A. Issues of Unequal Access to Public Health in India. Front Public Heal. 2015;3: 1–3. doi: 10.3389/fpubh.2015.00245 26579507 PMC4621381

[7] Vlahov D, Galea S, Freudenberg N. The Urban Health “Advantage.” J Urban Heal. 2005;82: 1–4. doi: 10.1093/jurban/jti001 15738341 PMC3456628

[8] McFarlane C, Silver J, Truelove Y. Cities within cities: intra-urban comparison of infrastructure in Mumbai, Delhi and Cape Town. Urban Geogr. 2017;38: 1393–1417. doi: 10.1080/02723638.2016.1243386

[9] Kumar A, Mohanty SK. Intra-urban differentials in the utilization of reproductive healthcare in India, 1992–2006. J Urban Heal. 2011;88: 311–328. doi: 10.1007/s11524-010-9532-7 21331748 PMC3079032

[10] Mberu BU, Haregu TN, Kyobutungi C, Ezeh AC. Health and health-related indicators in slum, rural, and urban communities: A comparative analysis. Glob Health Action. 2016;9: 1–13. doi: 10.3402/gha.v9.33163 27924741 PMC5141369

[11] Singh A, Kumar A, Pranjali P. Utilization of maternal healthcare among adolescent mothers in urban India: Evidence from DLHS-3. PeerJ. 2014;2014: 1–29. doi: 10.7717/peerj.592 25392750 PMC4226640

[12] Paria B, Bhattacharyya A, Das S. A comparative study on menstrual hygiene among urban and rural adolescent girls of West Bengal. J Fam Med Prim Care. 2014;3: 413. doi: 10.4103/2249-4863.148131 25657955 PMC4311354

[13] Singh A, Chakrabarty M, Chowdhury S, Singh S. Exclusive use of hygienic menstrual absorbents among rural adolescent women in India: A geospatial analysis. Clin Epidemiol Glob Heal. 2022;17: 101116. doi: 10.1016/j.cegh.2022.101116

[14] Dasgupta A, Sarkar M. Menstrual hygiene: How hygienic is the adolescent girl? Indian J Community Med. 2008;33: 77. doi: 10.4103/0970-0218.40872 19967028 PMC2784630

[15] Anand E, Singh J, Unisa S. Menstrual hygiene practices and its association with reproductive tract infections and abnormal vaginal discharge among women in India. Sex Reprod Healthc. 2015;6: 249–254. doi: 10.1016/j.srhc.2015.06.001 26614609

[16] Roeckel S, Cabrera-Clerget A, Yamakoshi B. Guide to menstrual hygiene materials. UNICEF. 2019; 6–36. Available: https://www.unicef.org/media/91346/file/UNICEF-Guide-menstrual-hygiene-materials-2019.pdf

[17] van Eijk AM, Sivakami M, Thakkar MB, Bauman A, Laserson KF, Coates S, et al. Menstrual hygiene management among adolescent girls in India: a systematic review and meta-analysis. BMJ Open. 2016;6: e010290. doi: 10.1136/bmjopen-2015-010290 26936906 PMC4785312

[18] Anand E, Unisa S, Singh J. Menstrual Hygiene Management among Young Unmarried Women in India. Soc Sci Spectr. 2015;1: 20–31. Available: http://socialspectrum.in/index.php/sp/article/view/6

[19] International Institute for Population Sciences. National Family Health Survey—5 2019–21. Minist Heal Fam Welf Natl. 2020;361: 2.

[20] Austrian K, Kangwana B, Muthengi E, Soler-Hampejsek E. Effects of sanitary pad distribution and reproductive health education on upper primary school attendance and reproductive health knowledge and attitudes in Kenya: a cluster randomized controlled trial. Reprod Health. 2021;18: 1–13. doi: 10.1186/s12978-021-01223-7 34465344 PMC8406733

[21] Misra P, Upadhyay RP, Sharma V, Anand K,… A community-based study of menstrual hygiene practices and willingness to pay for sanitary napkins among women of a rural community in northern India. Natl Med J India. 2013. Available: https://www.academia.edu/download/46698474/A_community-based_study_of_menstrual_hyg20160622-30028-bktxm5.pdf 25073990

[22] Bs DAM, Swarnalatha N, Devi BS, Swarnalatha N. Prevalence of RTI/STI among reproductive age women (15–49 YEARS) in urban slums of Tirupati Town, Andhra Pradesh. Heal Popul Perspect Issues. 2007;30: 56–70.

[23] Jessima S. Prevalence of urinary tract infection among school going adolescent girls in rural part of Chennai. Int Arch Integr Med. 2019;6: 278–282.

[24] Sivakami M, van Eijk AM, Thakur H, Kakade N, Patil C, Shinde S, et al. Effect of menstruation on girls and their schooling, and facilitators of menstrual hygiene management in schools: Surveys in government schools in three states in India, 2015. J Glob Health. 2019;9. doi: 10.7189/jogh.09.010408 30546869 PMC6286883

[25] Juyal R, Kandpal SD, Semwal J. Menstrual hygiene and reproductive morbidity in adolescent girls in Dehradun, India. Bangladesh J Med Sci. 2014;13: 170–174. doi: 10.3329/bjms.v13i2.14257

[26] Omidvar S, Amiri FN, Bakhtiari A, Begum K. A study on menstruation of Indian adolescent girls in an urban area of South India. Journal of family medicine and primary care. 2018. pp. 698–702. doi: 10.4103/jfmpc.jfmpc_258_17 30234040 PMC6132001

[27] Torondel B, Sinha S, Mohanty JR, Swain T, Sahoo P, Panda B, et al. Association between unhygienic menstrual management practices and prevalence. BMC Infect Dis. 2018;18: 473. Available: https://bmcinfectdis.biomedcentral.com/articles/10.1186/s12879-018-3384-2 30241498 10.1186/s12879-018-3384-2 PMC6150969

[28] Das P, Lisnek D, Sahoo KC, Sinha S, Mohanty J, Sahoo P, et al. Identifying risk factors for lower reproductive tract infections among women using reusable absorbents in Odisha, India. Int J Environ Res Public Health. 2021;18: 1–15. doi: 10.3390/ijerph18094778 33947145 PMC8124764

[29] Santhanakrishnan I, Athipathy V. Impact of health education on menstrual hygiene: An intervention study among adolescent school girls. Int J Med …. 2018. Available: https://www.researchgate.net/profile/Varshini-Athipathy-2/publication/324095234_Impact_of_health_education_on_menstrual_hygiene_An_intervention_study_among_adolescent_school_girls/links/5be289ef4585150b2ba460a5/Impact-of-health-education-on-menstrual-hygi

[30] Pandit C, Singh AK, Parajuli A, Pandey A. Misconception and Effect of Menstruation (Chhaupadi) and Delivery on Women’s Health in Bajura, District Nepal. Eur J …. 2021. Available: https://www.nepjol.info/index.php/ejms/article/view/39103

[31] Thakre SB, Thakre SS, Reddy M, Rathi N, Pathak K, Ughade S. Menstrual hygiene: Knowledge and practice among adolescent school girls of Saoner, Nagpur District. J Clin Diagnostic Res. 2011;5: 1027–1033. Available: https://www.ircwash.org/sites/default/files/Thakre-2011-Menstrual.pdf

[32] Ram U, Pradhan MR, Patel S, Ram F. Factors associated with disposable menstrual absorbent use among young women in India. Int Perspect Sex Reprod Health. 2020;46: 223–234. doi: 10.1363/46e0320 33108760

[33] Roy A, Paul P, Saha J, Barman B, Kapasia N, Chouhan P. Prevalence and correlates of menstrual hygiene practices among young currently married women aged 15–24 years: an analysis from a nationally representative survey of India. Eur J Contracept Reprod Heal Care. 2021;26: 1–10. doi: 10.1080/13625187.2020.1810227 32938257

[34] Maharana B. What Explains the Rural‑Urban Gap in the Use of Hygienic Methods of Menstrual Protection among Youth in the East Indian State of Bihar? Indian J Community Med. 2022;47: 182–186. doi: 10.4103/ijcm.ijcm_923_21 36034242 PMC9400350

[35] Wagh R V., Upadhye AJ, Upadhye JJ. Menstrual hygiene practices in young girls of urban India. Int J Reprod Contraception, Obstet Gynecol. 2018;7: 1897–1902. doi: 10.18203/2320-1770.ijrcog20181925

[36] Sonowal P, Talukdar K. Menstrual hygiene knowledge and practices amongst adolescent girls in urban slums of Dibrugarh town-a cross sectional study. Galore Int J Heal Sci Res. 2019;4: 44–51. Available: https://www.gijhsr.com/GIJHSR_Vol.4_Issue.1_Jan2019/9.pdf

[37] Kusuma YS, Pal M, Babu B V. Health insurance: Awareness, utilization, and its determinants among the urban poor in Delhi, India. J Epidemiol Glob Health. 2018;8: 69–76. doi: 10.2991/j.jegh.2018.09.004 30859791 PMC7325807

[38] Omidvar S, Begum K. Factors influencing hygienic practices during menses among girls from south India- A cross sectional study. Int J Collab Res Intern Med Public Heal. 2010;2: 411–423. Available: https://core.ac.uk/download/pdf/231804927.pdf

[39] Malhotra A, Coli S, Coates S, Mosquera-Vasquez M. Factors associated with knowledge, attitudes, and hygiene practices during menstruation among adolescent girls in Uttar Pradesh. Waterlines. 2016;35: 277–305. doi: 10.3362/1756-3488.2016.021

[40] Kathuria B, Raj TPS. Effects of socio-economic conditions on usage of hygienic method of menstrual protection among young women in EAG states of India. Amity J Healthc Manag. 2018. Available: https://amity.edu/UserFiles/admaa/0d862Paper 4.pdf

[41] Vishwakarma D, Puri P, Sharma SK. Interlinking menstrual hygiene with women’s empowerment and reproductive tract infections: evidence from India. Clin Epidemiol Glob Heal. 2020;10: 100668. doi: 10.1016/j.cegh.2020.11.001

[42] Muralidharan A, Patil H, Patnaik S. Unpacking the policy landscape for menstrual hygiene management: implications for school Wash programmes in India. Waterlines. 2015. Available: https://www.jstor.org/stable/24688193

[43] Rajagopal S, Mathur K. ‘Breaking the silence around menstruation’: experiences of adolescent girls in an urban setting in India. Gend Dev. 2017;25: 303–317. doi: 10.1080/13552074.2017.1335451

[44] McCammon E, Bansal S, Hebert LE, Yan S,… Exploring young women’s menstruation-related challenges in Uttar Pradesh, India, using the socio-ecological framework. Sex Reprod Heal Matters. 2020;28: 291–302. doi: 10.1080/26410397.2020.1749342 32308152 PMC7175471

[45] Nayaju R. “Chhaupadi”, Bringing Awareness on Menstruation Taboo Using Design Strategy. search.proquest.com; 2021. Available: https://search.proquest.com/openview/b322f0401472470bc06ed28a42204d47/1?pq-origsite=gscholar%5C&cbl=18750%5C&diss=y

[46] Dahlqvist A. It’s Only Blood: Shattering the Taboo of Menstruation. books.google.com; 2018. Available: https://books.google.com/books?hl=en%5C&lr=%5C&id=Rf80EAAAQBAJ%5C&oi=fnd%5C&pg=PP1%5C&dq=%22menstrual+health%22%7C%22menstruation%22%7C%22mnetsrual+hygiene+management%22%7C%22sanitary+napkins%22%7C%22hygienic+absorbents%22+%22india%22%5C&ots=8hB5tyFBNh%5C

[47] Sommer M, Sutherland C, Chandra-Mouli V. Putting menarche and girls into the global population health agenda. Reprod Health. 2015. doi: 10.1186/s12978-015-0009-8 25889785 PMC4396832

[48] Anton B., Kim W., Nair A., and Wang E. Menstrual Hygiene Management- Evidence from the 6th Round of MICS. Data Anal Sect Div Data, Anal Plan Monit UNICEF, New York. 2021.

[49] Ray S, Ghosh T, Mondal PC, Basak S,… Knowledge and information on psychological, physiological and gynaecological problems among adolescent schoolgirls of eastern India. Ethiop J …. 2011. Available: https://www.ajol.info/index.php/ejhs/article/view/73757 22434998 PMC3275869

[50] Bhatia D, Bhatia P, Chaudhary A, Anand BK,… Assessment of knowledge regarding menstruation, menstrual hygiene and various myths and taboos faced by the Adolescent Girls of Khera Khurumpur a small village in India. Eur J …. 2021. Available: https://ejmcm.com/article_9961_151.html

[51] Mason L, Sivakami M, Thakur H, Kakade N,… “We do not know”: a qualitative study exploring boys perceptions of menstruation in India. Reprod Health. 2017. doi: 10.1186/s12978-017-0435-x 29216895 PMC5721687

[52] Sommer M. Ideologies of sexuality, menstruation and risk: girls’ experiences of puberty and schooling in northern Tanzania. Cult Heal \&sexuality. 2009. doi: 10.1080/13691050902722372 19326264

[53] Chauhan S, Kumar P, Marbaniang SP, Srivastava S, Patel R, Dhillon P. Examining the predictors of use of sanitary napkins among adolescent girls: A multi-level approach. PLoS One. 2021;16: 1–14. doi: 10.1371/journal.pone.0250788 33930035 PMC8087036

[54] Vos T, Lim SS, Abbafati C, Abbas KM, Abbasi M, Abbasifard M, et al. Global burden of 369 diseases and injuries in 204 countries and territories, 1990–2019: a systematic analysis for the Global Burden of Disease Study 2019. Lancet. 2020;396: 1204–1222. doi: 10.1016/S0140-6736(20)30925-9 33069326 PMC7567026

[55] Goli S, Sharif N, Paul S, Salve PS. Geographical disparity and socio-demographic correlates of menstrual absorbent use in India: A cross-sectional study of girls aged 15–24 years. Child Youth Serv Rev. 2020;117: 105283. doi: 10.1016/j.childyouth.2020.105283

[56] Omidvar S, Begum K. Menstrual pattern among unmarried women from south India. J Nat Sci Biol Med. 2011. Available: https://www.ncbi.nlm.nih.gov/pmc/articles/PMC3276009/ doi: 10.4103/0976-9668.92329 22346231 PMC3276009

[57] Wright RE. Logistic regression. Reading and understanding multivariate statistics. Washington, DC, US: American Psychological Association; 1995. pp. 217–244.

[58] StataCorp. 2019. Stata Statistical Software: Release 16. College Station, TX: StataCorp LLC.

[59] Redlands CESRI. ArcGIS Desktop: Release 10. 2011.

[60] Marcoulides KM, Raykov T. Evaluation of Variance Inflation Factors in Regression Models Using Latent Variable Modeling Methods. Educ Psychol Meas. 2019;79: 874–882. doi: 10.1177/0013164418817803 31488917 PMC6713981

[61] India Today. Tripura govt to provide schoolgirls with free sanitary napkins. 21 Jan 2021. Available: https://www.indiatoday.in/education-today/news/story/tripura-govt-to-provide-schoolgirls-with-free-sanitary-napkins-1761327-2021-01-21#:~:text=The scheme involving the distribution,a period of three years.

[62] Navaneetham K, Dharmalingam A. Utilization of maternal health care services in Southern India. Soc Sci Med. 2002;55: 1849–1869. doi: 10.1016/s0277-9536(01)00313-6 12383469

[63] Singh S. How This Punjab School Changed The Way Boys Talk About Periods. 101Reporters. 2022. Available: https://101reporters.com/article/development/How_This_Punjab_School_Changed_The_Way_Boys_Talk_About_Periods

[64] Khanna A, Goyal RS, Bhawsar R. Menstrual Practices and Reproductive Problems: A Study of Adolescent Girls in Rajasthan. J Health Manag. 2005;7: 91–107. doi: 10.1177/097206340400700103

[65] Singh A, Kumar A, Kumar A. Determinants of neonatal mortality in rural India, 2007–2008. PeerJ. 2013;1: e75. doi: 10.7717/peerj.75 23734339 PMC3669267

[66] The World Bank. Population living in slums (% of urban population) —India. In: United Nations Human Settlements Programme [Internet]. 2018. Available: https://data.worldbank.org/indicator/EN.POP.SLUM.UR.ZS?locations=IN

[67] World Health Organization. Addressing Health of the Urban Poor in South-East Asia Region: Challenges and Opportunities. 2011. Available: https://apps.who.int/iris/rest/bitstreams/909415/retrieve

[68] De Schutter O. The Right to Adequate Housing. United Nations Human Settlements Programme. 2014. doi: 10.1057/dev.2014.64

[69] Centre on Housing Rights and Evictions (COHRE). Women, Slums, and Urbanization: Examining the Causes and Consequences. 2008. Available: https://www.alnap.org/system/files/content/resource/files/main/women-slums-and-urbanisation-may-2008.pdf

[70] Chakravarthy V, Rajagopal S, Joshi B. Does Menstrual Hygiene Management in Urban Slums Need a Different Lens? Challenges Faced by Women and Girls in Jaipur and Delhi. Indian J Gend Stud. 2019;26: 138–159. doi: 10.1177/0971521518811174

[71] Tamil Nadu Urban Sanitation Support Programme. Shame, superstition and secrecy hurting menstrual hygiene, finds Tamil Nadu study. scroll.in. 2022: 1–9. Available: https://scroll.in/article/910402/shame-superstition-and-secrecy-are-coming-in-the-way-of-menstrual-hygiene-finds-tamil-nadu-study

[72] Geertz A, Iyer L, Kasen P, Mazzola F, Peterson K. Menstrual Health in India | Country Landscape Analysis. Reprod Health Matters. 2016;51: 1–25. Available: https://menstrualhygieneday.org/wp-content/uploads/2016/04/FSG-Menstrual-Health-Landscape_India.pdf

[73] Kuruvilla A. ‘She Pad’ project in Kerala schools going strong- The New Indian Express. The New Indian Express. 2022. Available: https://www.newindianexpress.com/states/kerala/2019/jan/09/she-pad-project-in-schools-going-strong-1922561.html

[74] Express News Service. Gujarat govt to provide pack of 6 sanitary pads at Re 1. The Indian Express. 2022. Available: https://indianexpress.com/article/cities/ahmedabad/gujarat-govt-to-provide-pack-of-6-sanitary-pads-at-re-1-2773606/

[75] Department of Health and Family Welfare. Khusi. In: Odisha State Medical Corporation, Government of Odisha [Internet]. 2022. Available: https://khushi.nic.in/

[76] Gupta A. Free sanitary pads for girls in middle and high schools of Bihar. In: Down to earth [Internet]. Available: https://www.downtoearth.org.in/news/free-sanitary-pads-for-girls-in-middle-and-high-schools-of-bihar-44635#:~:text=Adolescent girl students enrolled in,for the past one year.

[77] Karan C. Rajasthan Menstrual Hygiene Scheme (MHS) | Muft Sanitary Pad Yojana 2021 | Free Sanitary Napkins for Women. Sarkariyojana. 2021. Available: https://sarkariyojana.com/rajasthan-menstrual-hygiene-scheme-mhs-sanitary-napkins-women/

[78] Pandey A. Under Swechha, Andhra govt to provide free sanitary napkins to school-going girls. In: India Today [Internet]. 5 Oct 2021. Available: https://www.indiatoday.in/india/story/swechha-andhra-govt-free-sanitary-napkins-school-college-girls-1860841-2021-10-05

[79] Sarkar S. Scheme to give free sanitary napkins to 22.50 lakh BPL women, girls launched. Hindustan Times. 6 Aug 2020. Available: https://www.hindustantimes.com/chandigarh/scheme-to-give-free-sanitary-napkins-to-22-50-lakh-bpl-women-girls-launched/story-4yPwwjdxpdGd8DfT87ZBEN.html

[80] Singh N. One-rupee sanitary pads welcome, but govt’s Janaushadhi stores often don’t have them. The Print. 2019: 283474. Available: https://theprint.in/india/one-rupee-sanitary-pads-welcome-but-govts-janaushadhi-stores-often-dont-have-them/283474/

[81] Sharma S. Taxing menstrual products is like taxing women for being women. Down to Earth. 2017. Available: https://www.downtoearth.org.in/news/health/taxing-menstrual-products-is-like-taxing-women-for-being-women-57859

[82] Kumar S. Menstrual health in India needs more than just distribution of low cost sanitary pads | ORF. Health Express: Observer Research Foundation. 2020. Available: https://www.orfonline.org/expert-speak/menstrual-health-in-india-needs-more-than-just-distribution-of-low-cost-sanitary-pads/

[83] Ministry of Chemical and Fertilizers. Sanitary Napkins available for Rs. 1 /—per pad at Pradhan Mantri Bhartiya Janaushadhi Kendras. 2020. Available: https://pib.gov.in/PressReleasePage.aspx?PRID=1632082

[84] Ministry of Chemical and Fertilizers. Janaushadhi Suvidha Sanitary Napkin will now be available at One Rupee per pad. 2019. Available: https://pib.gov.in/PressReleaseIframePage.aspx?PRID=1583141

[85] Thawani V, Mani A, Upmanyu N. Why the Jan Aushadhi scheme has lost its steam in India? J Pharmacol Pharmacother. 2017;8: 134–136. doi: 10.4103/jpp.JPP_38_17 29081624 PMC5642129

[86] UNICEF. Country Snapshot: Menstrual hygiene management in schools in South Asia: India. 2018; 1–4.

[87] MacRae ER, Clasen T, Dasmohapatra M, Caruso BA. ’It’s like a burden on the head’: Redefining adequate menstrual hygiene management throughout women’s varied life stages in Odisha, India. PLoS One. 2019;14: 1–23. doi: 10.1371/journal.pone.0220114 31369595 PMC6675075

