# Supplementary material for: Intra-urban differentials in the exclusive use of hygienic methods during menstruation among young women in India
Source: PLOS Glob Public Health. 2023 Jun 13;3(6):e0002047. doi: 10.1371/journal.pgph.0002047 (PMC10263343; doi:10.1371/journal.pgph.0002047)
Supplement: Attachment — Submitted filename: 221113_PGPH response to reviewer_Final.docx [file pgph.0002047.s001.docx]

*Dear Dr Johannes Stortz*

*We want to thank you for providing us with an opportunity to revise our paper entitled* ***“Intra-urban differentials in the exclusive use of hygienic methods during menstruation among young women in India” (PGPH-D-22-01066****). We would also extend our heartfelt thanks to reviewers for their valuable time and helpful comments and suggestions. The manuscript has been revised in light of the concerns raised in the review report. We have addressed all the issues raised and incorporated all the comments and suggestions made by the reviewers. Our point-to-point response to the comments, on which the reviewers needed further clarification from us or suggested modifications in the manuscript, is given below.*

**REVIEWER(S)’ COMMENTS TO THE AUTHOR**

**Reviewer 1**

Comment #1: The paper investigated the pattern of hygienic absorbents among menstruating women in urban India. The analysis is done and presented rigorously. The discussion section is excellent with all govt programs on the topic. However, if they can include slum and non-slum variables in their analysis to see the intra-urban difference, the quality of the paper will be improved.

Response #1: Thank you so much for your kind words. We really appreciate you taking the time out to review our paper.

Undoubtedly, including slum and non-slum factors would strengthen our paper. However, we would like to draw your attention to the fact that neither slum nor non-slum variables are included in the NFHS-5 dataset. We were therefore unable to include them into the analysis.

We have included this point as a limitation of our study and mentioned this in the discussion section.

**Reviewer 2**

The study is based on the latest data set of NFHS-5 and highlights few on the novel points in the mensural hygiene research domain. The statistical methods used are popular and unambiguous. The manuscript however contains a few factual errors and omissions in the text, however amendable. I would recommend it for publication with the following corrections.

Comment #1: The study missed citing some of previous studies on this subject from India. I suggest authors to review those studies and nuance their discussion section.

Goli, S., Sharif, N., Paul, S., & Salve, P. S. (2020). Geographical disparity and socio-demographic correlates of menstrual absorbent use in India: A cross-sectional study of girls aged 15–24 years. Children and Youth Services Review, 117, 105283.

Malhotra, A., Goli, S., Coates, S., & Mosquera-Vasquez, M. (2016). Factors associated with knowledge, attitudes, and hygiene practices during menstruation among adolescent girls in Uttar Pradesh. Waterlines, 35. <https://doi.org/10.3362/1756-3488.2016.021>.

Also, add some discussion on why the question of mensural hygiene for urban setting is important. What urban processes are pushing back the young girls in accessing and using mensural absorbents. In particular, the role of housing and access to WASH facilities.

Response #1: Thank you for the suggestion. We have reviewed the above-mentioned articles, and cited them in our study.

As per reviewer's suggestion, we have included a paragraph (line no 309-319) explaining why the issue of menstrual hygiene in urban settings is important, including inadequate housing and a lack of WASH facilities.

Comment #2: Line 31: The idea of an urban advantage in health and healthcare should be elaborated and a few references on it might be helpful.

Response #2: Thank you for the suggestion. As per reviewer’s suggestion we have elaborated the idea of urban advantage in health with few references (see line 37-39).

Comment #3: Line 74: The authors should justify the reason behind limiting the research to 15-24 age group.

Response #3: We have justified the reason behind limiting the research to 15-24 age group (see line 81-89).

Comment #4: Line 96: The authors have probably calculated Variance Inflation Factors (VIF) but refer it as Variance Influence Factors (VIF). If it’s just a typographical error it should be corrected, otherwise, they should elaborate the idea of Variance Influence Factors (VIF).

Response #4: Thank you for pointing this out. We have rectified the typographical error in our revised manuscript.

Comment #5: Line 105: The sentence in the line seems to be incomplete and has a misplaced period sign. This should be rewritten.

Response #5: Thank you for pointing out the issue. We have rewritten the sentence to convey our message clearly.

Comment #6: Line 124: “Women using both hygienic and unhygienic absorbents” in different spells are coded as "users of unhygienic absorbents". Is there any way possible to segregate these two categories if yes it may be disaggregated?

Response #6: Thanks for your suggestion. However, the objective of this study is to examine differentials in the **exclusive use** of hygienic absorbents among young women. As our focus is solely on the **exclusive use** of hygienic absorbents, that is why we created a binary variable, where we coded ‘exclusive users of hygienic absorbents’ as 1 (yes) and the rest of the population which includes both women, who use unhygienic absorbents or a combination of hygienic and unhygienic absorbents, as 0 (no).

Comment #7: Line 126: The authors should include some variables form NFHS data that measure the women access/barriers in getting hygienic absorbents for examples “distance travelled to buy sanitary napkins”.

Response #7: Thank you for this suggestion. It would have been interesting to explore how distance travelled to buy sanitary napkins can be a barrier to accessibility to hygienic absorbents. However, in our study, we could not include this because NFHS-5 data does not provide any such variable (“distance travelled to buy sanitary napkins”) measuring the women’s access to hygienic absorbents.

Comment #8: Line 183: The sentence “At the district level, urban adolescent women have more geographical diversity.” looks incomplete and does not add any information it must be corrected.

Response #8: Thanks for pointing this out. We have rewritten the sentence for better clarity. We hope it is clear now.

Comment #9: Line 284: The study shows perfect 100% use of hygienic absorbents by the women in the Siddipet district of Telangana state. This seems to an outlier in the study and must be explained in a couple of subsequent lines in the text.

Response #9: In the revised manuscript, we have edited this part of our discussion where numeric data (such as Siddhipet district) was directly being quoted from the results section. Instead, we now focus on major geographical patterns and attempt to explain them.

As for Siddhipet, it was an outlier. We did try and find out the reason behind very high exclusive use of hygienic methods in Siddhipet. Although, no scientific study has been conducted to study the reason for such a high percentage, going through newspapers and government websites, we could discern that Siddhipet was primarily an urban district with strong programmatic interventions by the state government and many large scale non-governmental initiatives. Recently, the government there is promoting use of menstrual cup and distributing them for free through Anganwadis and Councillors.

While revising the text in the discussion section, we thought it would be better to remove this from the discussion section as it was a one-off district for which it was difficult to find an explanation in academic literature. In the discussion section of the revised manuscript, therefore, we instead have placed more emphasis on other more relevant spatial patterns.

Comment #10: Figure 3.b: Some part of the map is kept blank if its due to unavailability of the data or for any other reasons the details should be given in a note.

Response #10: Thanks for raising this issue. We added a category named "no data available” to the map's legend, to represent the blank areas of the map.

Comment #11: Table 3 and 4: In both of these table the p-values has been specified up to two decimal points, these should be corrected, and authors should specify these values up to three decimal points.

Response #11: In table 3 and 4 we have specified the p-values up to three decimal points as per your suggestion.

**Reviewer 3**

This manuscript uses data from the NFHS 5 to describe associations between socio-demographic characteristics and the exclusive use of commercial menstrual products (termed 'hygienic absorbents' by the authors) compared to the non-exclusive use of these products and use of other improvised absorbents such as cloth.

I have five key concerns, noted below.

Comment #1: Multiple, very similar analyses and papers have been published on this same research question using the NFHS-4 data. The authors have not addressed these past studies in the background; indeed they claim that only small scale studies have been done.

A very quick google-scholar search will show multiple papers on the NFHS 4 data

Here's a few (I expect there are more):

Avijit Roy, Pintu Paul, Jay Saha, Bikash Barman, Nanigopal Kapasia & Pradip Chouhan (2021) Prevalence and correlates of menstrual hygiene practices among young currently married women aged 15–24 years: an analysis from a nationally representative survey of India, The European Journal of Contraception & Reproductive Health Care, 26:1, 1-10, DOI: 10.1080/13625187.2020.1810227

Kathuria, B., & TP, S. R. (2022). Factors Explaining Regional Variations in Menstrual Hygiene Practices among Young Women in India: Evidence from NFHS-4.

Maharana, B. (2022). What explains the rural-urban gap in the use of hygienic methods of menstrual protection among Youth in the East Indian state of Bihar? Indian Journal of Community Medicine, 47(2), 182.

Vishwakarma, D., Puri, P., & Sharma, S. K. (2021). Interlinking menstrual hygiene with Women's empowerment and reproductive tract infections: Evidence from India. Clinical Epidemiology and Global Health, 10, 100668.

Ram, U., Pradhan, M. R., Patel, S., & Ram, F. (2020). Factors associated with disposable menstrual absorbent use among young women in India. International Perspectives on Sexual and Reproductive Health, 46, 223-234.

This study is the first I've seen on the NFHS-5 data, which is more recent that the NFHS-4. However, the authors then need to justify what THIS study would add to our understanding of menstrual health and hygiene, and how it relates to the myriad of past studies on this exact same research question using the previous survey.

I am very concerned that the authors haven't contextualized this study in this past work.

Response #1: Thanks for your valuable suggestion. We agree that there are some published articles on this same research question using the NFHS-4 data. In our revised manuscript we have included and gave reference of these studies and tried to contextualise this study in the past work as suggested.

However, all the above-mentioned studies had considered both rural and urban sample combined. None of them has tried to investigate intra-urban differences in the exclusive use of hygienic absorbent among young women in India at national level. Our study is unique and adding to the existing knowledge in 3 aspects -

Firstly, this is the first national level study to use the urban sample to identify within urban area the disadvantaged groups among young women.

Secondly, in our study we have investigated the intra-urban disparities across 707 districts of India and identified the districts with low and ultra-low exclusive use of hygienic methods. Thus, paper provides understanding of geographical disparities in exclusive use of hygienic methods. Since district is a planning unit, the results provided in this report may be useful for policymakers in identifying the target districts to improve exclusive use among young urban women.

Thirdly, this study uses NFHS-5 data which for the first time also includes data on the use of menstrual cups. Thus, it provides a more comprehensive pictures of use of hygienic methods during menstruation.

Fourthly, NFHS-5 is the latest data available in the country and hence, the results obtained in this study are representing the latest situation of the exclusive use of hygienic absorbents among young women in urban India.

Comment #2: There is (necessary) increasing emphasis in menstrual health research on the importance of product choice. UNICEF's 2019 report on menstrual health and hygiene, and the supplement on menstrual materials outlines this quite well. Indeed, authors should note that the JMP MICS reports have reported national level information on menstrual product use - their indicator includes cloth as absorbent, recognizing this is the preference of some women.

This isn't to say the authors should do the same in the analysis, but that any manuscript needs to engage with this thinking and highlight WHY we would be interested only in commercial product use, in contrast to women having enough menstrual materials, or preferred materials.

Along these lines, I find the position of the paper that the goal should be all women using commercial products, rather than that women have access to enough information to make informed decisions and access to sufficient menstrual materials. This is a point also outlined in the recent Global Menstrual Collective definition of Menstrual Health, and the WHO statement of menstrual health and rights.

Response #2: Thank you, sir, for this valuable suggestion. Yes, we do agree with you that product choice one important aspect of this issue. Women should be able to choose between the available options. Those who wish to use clothes, they should be able to use clothes and those wish to use commercial products they should be able to use commercial products.

We would like to humbly submit that the position of this paper is NOT that all women should use commercial products. Rather the position of this paper is that all women should be able to manage their menstruation in a hygienic manner. For that, we are advocating the use of ‘hygienic’ methods. They may or may not be commercially produced.

Following the suggestion, we have, in the discussion section of the revised manuscript, mentioned the following two issues: i) women having access to enough information to make informed decisions and ii) expanding access to a range of menstrual materials to ensure women are able to exercise their freedom of choice. (See line no. 374-384)

Comment #3: I know this is likely due to what was asked in the survey, but there doesn't seem to be any differentiation between commercially produced reusable menstrual pads, and disposables. With a view to the potential environmental and cost sustainability of reusable materials, it is unclear why these aren't differentiated.

It isn't clear what 'locally prepared napkins' refers to - are these reusable or does this refer to disposables manufactured locally? There are also reusables manufactured commercially at scale.

Response #3: Thank you, sir, for pointing this out. We agree that commercially manufactured ‘reusable menstrual pads’ and ‘disposable menstrual pads’ should be differentiated in light of environmental and economic sustainability in the contemporary context. However, we cannot include them in the analysis of this paper because there is no such question included in the National Family Health Survey.

NFHS does not provide any definition for 'locally prepared napkins' neither in the questionnaire not in the national or state reports. Therefore, we cannot say anything about what sort of napkins these napkins are – reusable or disposable.

Comment #4: In such a large data set any differences are going to be statistically significant. Given that the outcome is a non-rare event, the odds ratios are difficult to interpret in terms of how MEANINGFUL these differences between socio-demographic characteristics are. I am not convinced at the value of these differences for practice.

'Conceptual framework' for only socio-demographic predictors seems overstated.

In a few places authors interpret odds ratios as meaning some women are 'more likely' to use hygienic absorbents - this is not correct interpretation of an odds ratio. No risk ratios are presented.

In the discussion the authors do a great job of describing many different initiatives across India to support menstrual product access. Can the NSFH data not be used to evaluate whether these initiatives have resulted in increased uptake, given we know there is a baseline from survey 4, and now data from survey 5? Again, it is highly problematic that the article doesn't engage with the past work on this.

Response #4: Thank you, sir, for your suggestion. Considering the relatively large sample size, we have followed the reviewers' advice and refrained from drawing conclusions about the factors for which we have observed only minor variations.

We have removed the phrase ‘more likely’, and revised the text to convey the intended meaning.

We appreciate your feedback, sir, but it is worth mentioning that in the conceptual framework of the study we have not only considered demographic (respondent's current age, age at menarche, age at marriage), socio-economic factors (education level of respondent, household wealth status, type of home, social group, rreligion) but also spatial factors (region of residence), women empowerment related factors (working status of respondent, own a bank account, own a mobile phone), and factors related to information and services (exposure to mass media, discussion on menstrual hygiene with health care workers). The selection of variables was based on existing research on menstrual hygiene management.

Thank you, sir, for kind words. As for your question - *can the NSFH data not be used to evaluate whether these initiatives have resulted in increased uptake?* As far as our knowledge goes, the NFHS-5 data cannot be used to determine whether menstrual hygiene promoting programs (mentioned in the discussion section) have resulted in an increase in the use of hygienic methods. To assess the effect of a programme, we require programme data. NFHS 4 and 5 do not provide any data about these programmes. Both NFHS 4 and NFHS 5 are cross-sectional data sets, and the same women are not surveyed again; they are not longitudinal studies. We cannot assume NFHS-4 and NFHS-5 as baseline and endline surveys.

Comment #5: I am concerned at some selective use of evidence in the Background section. The authors state that use of 'unhygienic absorbents' results in RTIs and school absenteeism.

But the relationships between menstrual hygiene practices and needs and these outcomes is highly mixed. Indeed the Austrian study cited found no effect of a pad-distribution intervention on girls' absenteeism. A series of work by Das and Torondel in Odisha has also noted varied relationships between different menstrual hygiene practices and RTI.

We do hypothesize relationships between unmet menstrual health and hygiene needs and these outcomes, but the literature and relationships between these concepts is much more complex (and more researched) than the brief background provided here.

Again, I am not sure 'unhygienic absorbents' is fairly applied to all non-commercial product use.

Response #5: We have revised the text keeping mind your valuable comment and have cited Das and Torondel’s research paper in the revised manuscript.

As for ‘uncertain and complex relationship’ – yes, we agree that the evidence is mixed, and the relationship is a complex one. Therefore, in the revised manuscript, following your valuable suggestion, we have tried to include both kind of studies – the ones that have proven the relationship and those that have presented evidence against it. We acknowledge the complexity of the relationship and therefore modified accordingly.

‘Cloth’ and ‘others’ are the non-commercial products included in our study. We have classified them as "unhygienic" since many national level studies, including the NFHS-5 report (see page 115) use the same classification.

To qualify as a hygienic absorbent/material/method, cloth must be adequately sterilised, cleaned with soap and clean water, dried and stored in a hygienic place. This is generally not possible because of many reasons including lack of water and toilet facilities, shame and stigma attached with menstruation. Women often dry their menstrual clothes under other clothing, and not in direct sunlight. Microbial infections can occur when women cannot dry menstruation clothing in direct sunshine. The use of such a cloth as menstrual hygiene material is associated with abnormal vaginal discharge, urogenital infection, and skin irritations (UNICEF, 2019). It means using clothes is neither sanitary nor safe for women's reproductive health. Also, there is no information in the NFHS about how women clean, dry, and store their cloth used for absorption of menstrual blood. We therefore cannot assume that cloth is always a ‘hygienic’ absorbent. Hence, it is put under ‘unhygienic’ category.

**References:**

1. MacRae ER, Clasen T, Dasmohapatra M, Caruso BA. “It’s like a burden on the head”: Redefining adequate menstrual hygiene management throughout women’s varied life stages in Odisha, India. PLoS One. 2019. Available: <https://journals.plos.org/plosone/article?id=10.1371/journal.pone.0220114>
2. Shah SP, Nair R, Shah PP, Modi DK, Desai SA, Desai L. Improving quality of life with new menstrual hygiene practices among adolescent tribal girls in rural Gujarat, India. Reprod Health Matters. 2013;21: 205–213. doi:10.1016/S0968-8080(13)41691-9
3. Das S, Mallick A, Bharati P, Biswas S. Effects of menstrual characteristics, symptoms and hygiene-related practices on menstrual experience: A comparative study between freeholder and tenant adolescent schoolgirls of North 24 Parganas, West Bengal, India. Anthropologischer Anzeiger; Bericht uber die biologisch-anthropologische Literatur. 2020. pp. 183–193. doi:10.1127/anthranz/2020/1161
4. Shamima Y, Sarmila M, Nirmalya M, et al. Menstrual hygiene among adolescent school students: an indepth cross-sectional study in an urban community of West Bengal, India. Sudan J Public Health 2013;8:60–4.
5. Khanna A, Goyal RS, Bhawsar R. Menstrual practices and reproductive problems: a study of adolescent girls in Rajasthan. J Health Manag 2005;7:91–107
6. UNICEF (2019). Guide to Menstrual Hygiene Materials.

Once again, we appreciate the insightful suggestions and comments provided by the anonymous reviewers. With the reviewers' helpful suggestions, we were able to make important changes in the paper, which we feel have greatly improved the quality of the article. After incorporating all of the revisions recommended by the reviewers, we are hopeful that the revised manuscript is suitable to be considered for publication in your prestigious journal.

Sincerely yours,

Aditya Singh

Mahashweta Chakrabarty

Rakesh Chandra

Sourav Chowdhury

Shivani Singh
