# Supplementary material for: Intra-urban differentials in the exclusive use of hygienic methods during menstruation among young women in India
Source: PLOS Glob Public Health. 2023 Jun 13;3(6):e0002047. doi: 10.1371/journal.pgph.0002047 (PMC10263343; doi:10.1371/journal.pgph.0002047)
Supplement: Attachment — Submitted filename: 230326_PGPH response to reviewer.docx [file pgph.0002047.s002.docx]

*Dear Dr Richard Ali,*

*We want to thank you for providing us with an opportunity to revise our paper entitled* ***“Intra-urban differentials in the exclusive use of hygienic methods during menstruation among young women in India” (PGPH-D-22-01066****). We would also extend our heartfelt thanks to reviewers for their valuable time and helpful comments and suggestions. The manuscript has been revised in light of the concerns raised in the review report. We have addressed all the issues raised and incorporated all the comments and suggestions made by Reviewer 3. Our point-to-point response to the comments, on which the reviewer needed further clarification from us or suggested modifications in the manuscript, is given below.*

**REVIEWER(S)’ COMMENTS TO THE AUTHOR**

**Reviewer 3**

The manuscript has been strengthened through author attention to the peer reviewer comments.

I appreciate the authors expanding attention in the background to disparities among urban populations. However, I do not feel all of the comments have been sufficiently addressed.

Comment#1 - there are still places in the manuscript that report odds ratios as if they are risk ratios and state that respondents were 'more likely' to have a particular outcome. This is an important misrepresentation of statistics and the manuscript cannot be published before this is addressed through out

e.g., page 8 "Urban women who got their first period after age 16 were slightly more likely to use hygienic methods than those who got their first period earlier."

What do we mean by 'slightly' more likely. In my first comments to the authors, I highlighted that any differences among the groups were likely to be statistically significant given the sample size. "More likely" appears again on page 13 and throughout other places.

Response#1 – Thank you for bringing to our attention the issue that we overlooked in our manuscript. We appreciate your feedback and have replaced this term (more likely) with appropriate words.

Thank you for bringing your concern regarding the sentence on page 8 to our attention. We have made necessary revisions to enhance the clarity of the sentence, which now reads as follows: "The exclusive use of hygienic methods is comparatively higher among urban women who experienced menarche after the age of 16, than among those who experienced menarche earlier." This revised sentence more clearly conveys the intended meaning.

Additionally, we acknowledge your point regarding the inappropriate reporting of odds ratios as risk ratios. In our revised manuscript, we have replaced the term "more likely" with the appropriate terminology, as per your suggestion. The revised language can be found on pages 11-12, lines 261-286.

Thank you again for your valuable input, which has helped us to improve the rigor and clarity of our research.

Comment#2 - The authors acknowledged in their reply that the relationship between menstrual material use and urogenital infection/RTI is complex and under-researched. Authors have added some citations around this in the paper but have still included strong statements assuming that the use of cloth (as defined as 'unhygenic') causes negative outcomes.

This is present even in the revised abstract "The use of hygienic methods during menstruation is substantially lower among urban women in India than in many other developing countries, resulting in higher reproductive morbidity." As highlighted in my past review, and acknowledged by the authors, this statement is not sufficiently supported by current evidence and should be tempered.

Response#2 – We appreciate the reviewer's insightful feedback on our manuscript. We agree with the reviewer's point that the relationship between menstrual material use and urogenital infection/RTI is complex and under-researched. Therefore, we have taken the reviewer's suggestion and replaced this sentence in the abstract with a suitable sentence as follows: “*Menstrual hygiene among women is a critical public health issue in urban India, but it remains understudied and under-researched.”*

Comment#3 - I understand that the authors want to use the same language as the NFHS - I still feel very uncomfortable that the authors are referring to cloth as 'unhygienic' - it evokes a judgement on women that I feel adds to the stigma around menstruation. As the authors note, cloth could be considered hygienic if cleaned appropriately. I suggest the authors consider if 'hygienic' is the language they want to use - and if so perhaps add some reflective text on this in the introduction.

Response#3 - We appreciate the valuable feedback provided by the reviewer regarding our paper. We acknowledge their concern regarding the classification of cloth as "unhygienic," as this could potentially contribute to the stigma surrounding menstruation.

While it is true that washing and drying menstrual cloth with soap and water and exposing them to direct sunlight can be considered a hygienic method, the NFHS data that we used for our study does not provide any information on the specific process used by women to wash, dry, and store their menstrual cloth. Therefore, we relied on the classification and language used by the NFHS.

To address the reviewer's concern, we have removed the term "unhygienic" from the paper altogether. We agree that the use of this term could potentially lead to negative judgments and stigmatization of women who use cloth during menstruation.

We have also added some reflective text as suggested by author. See Lines 58-64 Page 3 - “*Although the established classification scheme is suitable for analytical purposes, it has the potential limitation of not being able to capture the full range of women's experiences and perspectives regarding menstrual hygiene. It must be emphasized here that the use of this classification in this paper is solely intended for analytical purposes and is not intended to stigmatize or marginalize women who use alternative menstrual hygiene products. The authors are committed to using language that is precise, inclusive, and respectful of the diversity of women's experiences with menstrual hygiene.*”

We appreciate the reviewer's feedback, which has helped us to improve the clarity and sensitivity of our language in discussing menstrual hygiene practices. Thank you for your valuable input.

Comment#4 - Authors have responded to my comment about informed choice by including some mentions of this in the discussion. I can't see attention to understanding if women have ENOUGH menstrual materials. Notably research has found that use of commercial pads doesn't necessarily imply that women have enough materials.

<https://www.mdpi.com/1660-4601/17/8/2633>

This study found that across 10 countries, among respondents exclusively using pads 26% still reported having unmet menstrual material needs.

The limitations section of the manuscript should highlight not only the limitations of the cross-sectional design - but also be clear about what this data can and can't tell us about menstrual product access & menstrual health in India.

Response#4 – We appreciate the thoughtful feedback provided by the reviewer regarding the limitations of our study. Since we have utilized the NFHS data for our study, we acknowledge that this dataset does not contain information on unmet need of menstrual materials. To address the reviewer's concern, we have made the necessary revisions to the manuscript to more explicitly acknowledge this limitation in the limitations section of the discussion (see lines 395-402). We have also incorporated the reviewer's suggestions to improve the clarity and specificity of our language in discussing this issue.

*We have carefully considered all of the revisions recommended by the reviewer and have incorporated them into the revised manuscript. We believe that these changes have significantly improved the overall quality of the article and have addressed the concerns raised by the reviewer in a thorough and transparent manner.*

*As a result of the reviewer's valuable input, we are hopeful that the revised manuscript is now ready for consideration for publication in your esteemed journal. We thank the reviewer once again for their valuable time and effort in providing us with such helpful feedback.*

*Sincerely yours,*

*Aditya Singh*

*Mahashweta Chakrabarty*

*Rakesh Chandra*

*Sourav Chowdhury*

*Shivani Singh*
